# Microencapsulation of Red Sorghum Phenolic Compounds with Esterified Sorghum Starch as Encapsulant Materials by Spray Drying

**DOI:** 10.17113/ftb.57.03.19.6146

**Published:** 2019-09

**Authors:** Adriana García-Gurrola, Susana Rincón, Alberto A. Escobar-Puentes, Alejandro Zepeda, Fernando Martínez-Bustos

**Affiliations:** 1 National Technological Institute of Mexico/I.T. Mérida, Av. Tecnológico km 4.5 S/N, C.P. 97118 Merida, Yucatan, Mexico; 2 Autonomous University of Yucatan, Periferico Norte kilómetro 33.5, Chuburna de Hidalgo Inn, C.P. 97203 Mérida, Yucatan, Mexico; 3 Center for Research and Advanced Studies of the IPN, campus Queretaro Libramiento Norponiente 2000, Fracc. Real de Juriquilla, C.P. 76230 Santiago de Queretaro, Queretaro, Mexico

**Keywords:** microencapsulation, sorghum phenolic compounds, sorghum starch, esterification, extrusion

## Abstract

Phenolic compounds with antioxidant properties are highly sensitive molecules, which limits their application. In response, extruded esterified starch has been proposed as efficient encapsulating material. In this work, we aim to describe the encapsulation of red sorghum phenolic compounds by spray drying using extruded phosphorylated, acetylated and double esterified sorghum starch as wall material. Their respective encapsulation yields were 77.4, 67.4 and 56.8%, and encapsulation efficiency 91.4, 89.7 and 84.6%. Degree of substitution confirmed esterification of the sorghum starch and Fourier transform infrared spectroscopy showed the significant chemical and structural changes in the extruded esterified starch loaded with phenolic compounds. Microcapsules from phosphorylated sorghum starch showed the highest endothermic transition (173.89 °C) and provided a greater protection of the phenolic compounds during storage at 60 °C for 35 days than the other wall materials. Extruded esterified sorghum starch proved to be effective material for the protection of phenolic compounds due to its high encapsulation efficiency and stability during storage.

## INTRODUCTION

Sorghum is an important cereal grain worldwide due to its resistance to drought and its high starch content (70%); it has low production costs and multiple agroindustrial applications (*1*). In addition, it has been previously reported that pigmented testa of sorghum genotypes have high phenolic content (*2*) and hydroxybenzoic and hydroxycinnamic acids, in addition to being a unique source of 3-deoxyanthocyanidins, with high anticancer and antioxidant capacities (*3*). *In vitro* studies have shown that the exposure of cancerous cells of the colon and the oesophagus to polyphenolic sorghum extracts decreases or inhibits cell proliferation (*4*, *5*). However, although polyphenolic phytochemicals provide various positive health benefits, some problems related to their bioactivity are a cause for major concern during food processing, since they are sensitive to oxygen, temperature, pH, light, and gastrointestinal tract environment (*6*). Therefore, the efficacy, bioactivity and bioavailability of phenolic compounds will depend on their preservation and stability. In this respect, the encapsulation of phenolic compounds could help to address some of the previously mentioned disadvantages. Prior studies have demonstrated that the use of encapsulated polyphenols rather than free molecules enhances stability during storage (*7*). To achieve this, spray drying is regarded as one of the most promising techniques for the encapsulation and protection of certain classes of phenolic compounds (*8*), in that this is a highly efficient, economical process, and provides chemical stability to sensitive molecules such as phenolic compounds. Additionally, this process results in quality particles, and has been employed for the preparation of dry, stable food additives and flavours (*9*). Several classes of phenolics, such as anthocyanins from *Berberis vulgaris* (*10*), flavonoid extract from *Hypericum perforatum* (*11*), and polyphenols from *Rosmarinus officinalis* L. leaves (*12*), have been encapsulated by spray drying to improve the storage stability of phenolic compounds.

Different biopolymers, such as protein and polysaccharides, have served for the encapsulation of phenolic compounds by spray drying. It was previously reported that the encapsulation of gallic acid by spray drying with conventionally acetylated starch resulted in an efficiency and encapsulation yield of 62 and 74.3%, respectively (*13*). However, previous results demonstrated that esterified and extruded types of starch were effective encapsulation agents of hydrophobic (*14*) and hydrophilic (*15*) active ingredients with high efficiencies and encapsulation yields when spray drying was utilized.

Therefore, it is clear that sorghum grain is emerging as a potential source of polyphenolic compounds with health benefits, in addition to being a cereal with high starch content. To our knowledge, there is no published information on the esterification of white sorghum starch by reactive extrusion for evaluation as a wall material. Also to our knowledge, there are no reports regarding encapsulation of red sorghum phenolic compounds using starch derivatives as wall materials. Moreover, to date and to our knowledge, no double modification of the starch has been reported to obtain a phosphorylated and acetylated starch by extrusion. Based on the latter, we are raising the following as aims in this paper: (*i*) to develop three different esterified sorghum starch types by reactive extrusion, (*ii*) to evaluate the encapsulation of red sorghum phenolic compounds by spray drying, and (*iii*) to evaluate the storage stability of microencapsulated red sorghum phenolic compounds.

## MATERIALS AND METHODS

### Materials

A white sorghum grain cultivar ’Sinaloense’ (donated by Dr Pecina from the National Institute of Forestry, Agricultural and Livestock Research (INIFAP), Guanajuato, Mexico) was used for the isolation of starch according to the wet milling method (*16*). A red sorghum grain cultivar ’Pajarero’, kindly donated by INIFAP Mexico, was used for the extraction of phenolic compounds. Capsul® starch was purchased from Ingredion México S.A de C.V. (Guadalajara, Mexico), hydrochloric acid, sodium hydroxide, ethanol, potassium hydroxide, phenolphthalein, potassium bromide, gallic acid, methanol, acetic acid, Folin-Ciocalteu reagent, acetic anhydride and sodium tripolyphosphate (STPP) were acquired from Sigma-Aldrich Chemical Co., Merck (St Louis, MO, USA). All reagents were of analytical grade.

### Esterification of starch

#### Sample preparation

Native starch was hydrolyzed with HCl according to the method of Zambrano and Camargo (*17*) prior to esterification by reactive extrusion.

#### Phosphorylation of starch

Phosphorylation of starch was carried out with STPP by reactive extrusion based on the method of Chang and Lii (*18*). The previously hydrolyzed starch was conditioned with STPP (4 g/100 g). Briefly, the volume of distilled water necessary to adjust the moisture content to 18% was used to dissolve STPP and 0.1 M HCl on a magnetic stirrer (model FE-310; Felisa, Guadalajara, Jalisco, Mexico) for approx. 10 min. The pH of the solution was 4.5. The prepared solution was sprayed on starch and mixed vigorously with a laboratory spatula. Before extrusion, the conditioned starch was stored for 12 h at 4 °C in plastic bags to ensure a homogeneous water distribution.

#### Acetylation of starch

Acetylation was carried out by the extrusion process according to the method of Mali and Grossmann (*19*). The volume of 2.5 mL acetic anhydride was added to 100 g of hydrolyzed starch. The hydrolyzed starch was conditioned at 18% feed moisture and the pH adjusted to 8.5–9.0 with 0.1 M NaOH as previously mentioned. The obtained starch was stored for 12 h at 4 °C to ensure a homogeneous water distribution.

#### Double esterification of acetylated and phosphorylated starch

For double esterification, 2 g of STPP per 100 g hydrolyzed starch and 1.42 mL of acetic anhydride per 100 g of hydrolyzed starch were added simultaneously to the starch and conditioned to 18% moisture content and pH adjusted to 4.5–5.0 with 0.1 M HCl, as previously mentioned. The conditioned starch was stored for 12 h at 4 °C to ensure homogeneous water distribution.

#### Extrusion process

The conditioned starch samples were processed in a single screw extruder designed and manufactured by CINVESTAV-IPN (Querétaro, Mexico), with a 20-mm inner barrel diameter. Barrel temperatures were maintained at 60, 130 and 170 °C at the feeding, transition and high-pressure extrusion zones, respectively. Screw speed was 80 rpm and feed rate was 70 g/min, while the compression ratio of the screw was 3:1. The resulting extruded samples were dried in the oven (40 °C), milled and sieved through a mesh with a 149 µm pore size. Powder samples of esterified starch were packed for subsequent characterization and microencapsulation of phenolic compounds.

#### Degree of substitution of phosphorylated starch

Phosphorus content and degree of substitution (DS) in phosphorylated sorghum and double esterified sorghum starch were determined by the method of Smith and Caruso (*20*). Three repetitions were carried out for each analysis and the mean value was reported. DS was calculated as follows:

 DS=(162·*w*(P))/(3100–124·*w*(P)) /1/

where *w*(P) is the mass fraction of phosphorus (on dry mass basis) in the phosphorylated sorghum starch, 162 is the molar mass of the anhydroglucose unit, 3100 is the atomic mass of phosphorus multiplied by 100 and 124 is the molar mass of the phosphate substituent.

#### Determination of the amount of acetyl groups and degree of substitution

The amount of acetyl groups and the DS of the acetylated sorghum and double esterified sorghum starch were determined by titration, as described by Wurzburg (*21*). Acetylated starch (1 g) was placed in a 250-mL flask, and 50 mL of 75% ethanol in distilled water were added. The stoppered flask was covered with aluminium foil, placed in a water bath at 50 °C for 30 min and then cooled. Then, 40 mL of 0.5 M KOH were added and kept under constant stirring at 200 rpm (model FE-310; Felisa) for 72 h. After that, the excess of alkali was titrated with 0.5 M HCl, using phenolphthalein as an indicator. The original, unmodified starch served as control. The molality of acetyl groups (*b*/%) was calculated according to the following equation:

*b*(acetyl)=[(*V*_control_–*V*_sample_)·(*c*(HCl)+0.043·100)/*m*_sample_] /2/

where *V*_control_ and *V*_sample_ are the titration volumes for the blank and sample respectively (mL), *m*_sample_ is the sample mass (g), and 0.043 is the milliequivalent of the group CH_3_–C=O. DS was calculated as follows:

DS=[162·*b*(acetyl)/(4300–(42·*b*(acetyl))] /3/

where 162 is the molar mass of the anhydroglucose unit, 4300 is the atomic mass of group CH_3_–C=O multiplied by 100 and 42 is the atomic mass of group CH_3_–C=O minus 1.

#### Fourier transform infrared spectroscopy

Fourier transform infrared spectroscopy (FTIR) analyses were performed to determine the structural changes in derivatized starch samples (acetylated, phosphorylated and double esterified sorghum starch) compared to native starch with a spectrometer (GX PerkinElmer; Boston, MA, USA). The microparticles of the three types of starch were prepared by finely grinding the powder with KBr (1:100 *m*/*m*). The spectrum was recorded in a range between 400 and 4000 cm^–1^.

### Polyphenolic extract

Phenolic compounds were extracted from the red sorghum grain. A sample of finely ground sorghum grain (6.25 g) was stirred in 250 mL of acidified ethanol (0.01% (*V*/*V*) HCl in 70% ethanol) (pH=2.5). After 1 h of extraction, the solution was centrifuged (Hermle Z513 K equipment; Wehingen, Germany) for 10 min at 5000×*g* and filtered by vacuum filtration through a Whatman No. 1 filter paper to recover the solids. The process was repeated on the solids (a 250-mL volumetric flask containing 10 mL of acidified ethanol) and the extracts were combined. Total polyphenol content was estimated using the Folin-Ciocalteu method (*22*), and the results were expressed in mg of gallic acid equivalents (GAE) per g sample (dry mass basis) with a mass of 0–200 mg of gallic acid according to the calibration curve:

y=0.0031x+0.009; R^2^=0.9965 /4/

### Microencapsulation and characterization of microcapsules

The three different types of derivatized starch (phosphorylated, acetylated and double esterified starch) were used for microencapsulation of red sorghum polyphenols by spray drying (SD-Basic spray dryer; LabPlant, Huddersfield, UK). Emulsions of 10 g of starch esterified by extrusion, 60 mL of the polyphenolic extract and 30 mL of distilled water were prepared. The internal phase was added at 30 °C to form the emulsion immediately prior to drying. Emulsions were homogenized with an Ultra Turrax T-25-SI homogenizer (IKA Works, Wilmington, NC, USA) at 14 000 rpm for 5 min. The dispersion was maintained under constant stirring with a magnet bar during drying. Drying conditions were as follows: inlet air temperature (150±1) °C, outlet air temperature (80±5) °C, nozzle diameter 0.5 mm, and a liquid flow rate 10 mL/min. The airflow equipment was set at 70 m^3^/h. Dried powder microcapsules were collected from the base of the cyclone for subsequent characterization.

#### Efficiency and yield of encapsulation

Encapsulation efficiency (EE) and encapsulation yield (EY) were quantified by determining the concentration of superficial and total polyphenols (*23*). For determination of the concentration of superficial polyphenols, a sample (200 mg) of microcapsules was treated with 2 mL of ethanol and methanol (50:50); these dispersions were stirred in a vortex (Genius 3; IKA, Wilmington, NC, USA) for 1 min and then filtered (0.45 μm pore). For determination of total polyphenols, microcapsules (200 mg) were dispersed in 2 mL of methanol, acetic acid and water (50:8:42 *V*/*V*/*V*), stirred using a vortex mixer (Genius 3; IKA) for 1 min, ultrasonicated in a 20-kHz Branson ultrasonic bath (Dietzenbach, Germany) twice for 20 min, and then centrifuged at 112 000×*g* for 5 min. Finally, the residues of both total and superficial polyphenols were quantified according to the Folin-Ciocalteu method (*22*) as mentioned above.

EE and EY were determined according to the following equations:

EE=(*w*(total polyphenols–surface polyphenols))/ *w*(total polyphenols)·100 /5/

and

EY=*w*(total polyphenols in microcapsules)/  *w*(total polyphenols in emulsion)·100 /6/

### Differential scanning calorimetry

Differential scanning calorimetry (DSC) was used to investigate the thermal properties of microcapsules (loaded with sorghum polyphenols) of phosphorylated, acetylated, double esterified starch and Capsul® starch employing a DSC instrument (Mettler Toledo model 821; Schwerzenbach, Switzerland) calibrated with indium. Each sample (7 mg) was heated on an aluminium pan at a rate of 10 °C/min at between 25 and 210 °C.

### Scanning electron microscopy

The morphological characteristics of the microcapsules were evaluated using SEM (Philips XL30; Morrisville, NC, USA) at an acceleration voltage of 3 kV. Dried microcapsule samples were previously sprinkled on a double-sided sticky tape placed on aluminium stubs and covered with thin graphite film.

### Accelerated storage stability test

Microcapsules loaded with red sorghum phenolic compounds were stored at 60 °C in an oven with controlled temperature and in the absence of light for 35 days. Samples of 200 mg of microcapsules (phosphorylated, acetylated and double esterified starch) and Capsul® (as control wall material) were transferred into 100 mm×150 mm clear glass vials. Determination of total polyphenols was performed to evaluate changes during storage. Triplicate vials were removed every 7 days.

### Statistical analysis

All experiments were performed in triplicate. The differences between mean values of the treatments were determined using analysis of variance (ANOVA) and the paired Tukey’s test (p<0.05) with IBM SPSS v. 20 statistical software (*24*).

## RESULTS AND DISCUSSION

### Characterization of esterified starch

#### Degree of substitution of three starch samples

Sorghum starch was esterified by reactive extrusion and phosphorylated, acetylated and double esterified starch were obtained. Results of the degree of substitution (DS) were 0.040 and 0.043 for phosphorylated and acetylated starch, respectively, and 0.017 and 0.037 for double esterified starch (phosphate and acetyl groups, respectively) (data not shown). In this regard, the percentages of acetylation and phosphorylation were higher for acetylated and phosphorylated starch, respectively. Double esterified starch had greater number of hydroxyl groups substituted by the phosphate and acetyl groups than acetylated and phosphorylated starch. This could be explained by the transformation of the crystalline regions into amorphous regions when the acetic acid is mixed with starch at high temperature, contributing to the contact between the OH groups of the starch molecules and the substituent groups, in this case, the phosphate and acetyl groups (*25*). We compared the results for acetylated starch with those of Colussi *et al.* (*26*), who reported that a mass fraction of 5 g/100 g of starch resulted in a DS of 0.05, which was very close to that in the present work. However, these authors used a much higher amount of reagent, and a longer reaction time, so their results could be explained by severe extrusion conditions such as high temperature and shear forces, which cause starch granules to lose their granular structure and to open up. This facilitates exposure to the inter- and intramolecular hydroxyl groups of the starch, facilitating the formation of esterified acetyl groups. Finally, the results for phosphorylated starch are comparable to those of Seker and Hanna (*27*), who reported that the phosphate groups were able to bind to the starch in less than 2 min, which was attributed to the fact that during extrusion the starch granules break down physically. Thus, consequently, the water molecules and the phosphate groups easily penetrate the starch granules and improve the esterification rate.

#### FTIR spectra of starch samples

**Fig. 1:**
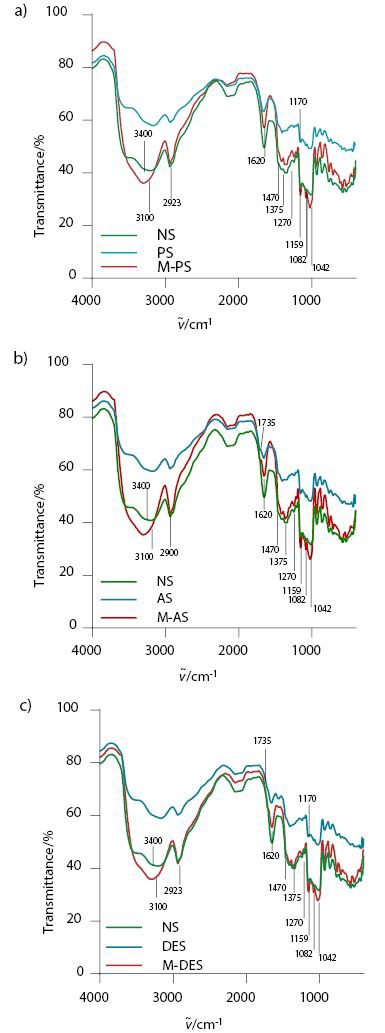
FTIR spectra of: a) phosphorylated starch (PS) and microcapsules from phosphorylated starch (M-PS) compared with those of native starch (NS), b) acetylated starch (AS) and microcapsules from acetylated starch (M-AS) compared with those of NS, and c) double esterified starch (DES) and microcapsules with double esterified starch (M-DES) compared with those of NS

The introduction of acetyl and phosphate groups into the starch molecule and the spectral changes that took place was confirmed by FTIR analysis (Fig. 1). Native starch showed the characteristic signals corresponding to the elongation of the main groups of the starch molecule as follows: 1470, 1270 and 1375 cm^–1^ corresponding to the carboxylic group, and 1159, 1082 and 1042 cm^–1^, assigned to the stretching of the carbonyl group of the glucose anhydride ring (*28*). However, these signals were found to decrease for the three starch derivatives developed by extrusion, which could be explained due to previous acid hydrolysis, as well as to the shear and high pressure inside the extrusion chamber, which broke the C–C and C–O bonds of the glycosylated molecule. The signal observed at 2923 cm^–1^ corresponds to the vibrations of the carbon hydrogen bonds of the methyl groups, the wide band at 3421 cm^–1^ is due to the stretching of the hydroxyl groups in the starch (*29*), while the OH groups appear with great intensity in native sorghum starch. However, in contrast to the results in Fig. 1, the intensity of this band was reduced in the three starch samples, which indicates the loss of OH groups due to the esterification with phosphate and acetyl groups in phosphorylated and acetylated starch, respectively.

Fig. 1a shows the changes resulting from the introduction of the phosphate groups into the starch, identified with a small but present signal in the 1170 cm^–1^ band. For acetylated starch (Fig. 1b), a new signal is observable in the band at 1735 cm^–1^, which is characteristic of the acetyl group modulus (*30*). In Fig. 1c, spectroscopic signals for the phosphate (1170 cm^–1^) and acetyl groups (1735 cm^–1^) of double esterified starch are observable; however, the signals are weaker, which is associated with the low DS obtained for each functional group (0.037 and 0.017, respectively). Nonetheless, these values show a greater degree of substitution (0.054) of hydroxyl groups.

#### Efficiency and encapsulation yield

Total polyphenolic content in the red sorghum grain as GAE was 30 mg/g (dry mass basis). This polyphenolic extract was used as an internal phase during microencapsulation. Encapsulation yield (EY) indicates the amount of polyphenols recovered in the encapsulation process, based on the initial amount of polyphenols added to the emulsion per g of starch against the amount of polyphenols recovered per g of capsules. Encapsulation efficiency (EE) indicates the potential of the encapsulating material to maintain the polyphenols inside the capsule (*31*), where the content of polyphenols within the capsule is considered in relation to the polyphenols on its surface. There were significant differences (p<0.05) in EY among all treatments; however, the three derivatized starch types used as encapsulating materials did not reveal significant differences in EE in relation to the control sample (capsule) (p>0.05) (Table 1). As can be observed in Table 1, phosphorylated starch had best results of EE and EY, followed by acetylated, and finally double esterified starch. The best results of EY and EE of microcapsules with phosphorylated starch are probably due to stronger interaction between the phenolic compounds and the encapsulating material through hydrogen bonds and van der Waals forces (*32*). This greater attraction of polyphenols in phosphate starch is due to the presence of phosphate groups and their delocalization property, which causes the oxygen to attract the protons of the OH group in phenols with more force, increasing the interaction between the wall materials and the polyphenolic compounds. This is in agreement with the degree of substitution, since greater substitution by the phosphate groups is in the phosphorylated than in double esterified starch, which makes the phosphorylated starch the best encapsulating material. On the other hand, the esterified acetyl groups in the starch molecules led to a more open structural reorganization due to steric hindrance and repulsion among molecules. This phenomenon led to the penetration of water to the amorphous regions of the starch (*33*) and resulted in a high formation of lumps, which rendered drying of the microcapsules difficult. It is noteworthy that the presence of lumps in double esterified and phosphorylated starch was minimal. For comparison, Robert *et al.* (*13*) reported an EY of 54, 65 and 74% for gallic acid microcapsules using native acetylated starch as wall materials with a DS of 0.096 and 0.19, respectively. Saikia *et al.* (*34*) encapsulated the polyphenolic extract of *Averrhoa carambola* pomace with maltodextrin as wall material by spray drying, and obtained EE values of 62.99, 74.1 and 79.07% in their treatments with polyphenol/maltodextrin at ratios 1:10, 1:15 and 1:20, respectively. Bušić *et al.* (*12*) encapsulated the polyphenols from *Rosmarinus officinalis* leaves with whey protein isolates and maltodextrins by spray drying and obtained EE ranging from 39.57 to 42.83%. Robert *et al.* (*13*) encapsulated gallic acid using different mixtures or native starch and acetylated starch by spray drying, and obtained EE ranging from 47 to 61%. As can be observed, the majority of authors obtained lower values ​​than those in this study. The differences are explained due to the fact that these authors used materials based on unmodified or acetylated polymers that do not have as high an attraction as that of the phosphate ion.

**Table 1:** Encapsulation yield and encapsulation efficiency of esterified sorghum starches as wall materials

Wall material	EY/%	EE/%
M-PS	(77.4±0.1)^a*^	(91.4±0.2)^a^
M-AS	(67.4 0.4)^b^	(89.7±0.2)^a^
M-DES	(56.8±0.3)^c^	(84.6±0.5)^b^
Capsule® (control)	(101.03±0.01)^d^	(88.1±0.10)^ab^

M-PS=microcapsules with phosphorylated starch, M-AS=microcapsules with acetylated starch, and M-DES=microcapsules with double esterified starch. Results show the mean value±standard error from three samples. Values with different lower-case superscript letters in the same column are significantly different from each other according to the Tukey’s test (p<0.05)

### Characterization of microcapsules with phenolic compounds

#### FTIR spectra of microcapsules

Fig. 1 corroborates the interaction of the polyphenolic compounds with the different microcapsules. FTIR spectra corresponding to all of the microcapsules: of phosphorylated starch (Fig. 1a), acetylated starch (Fig. 1b) and double esterified starch (Fig. 1c) had a stronger signal in the band at 3100 cm ^–1^ than the spectra of all of the starch esterified by extrusion (phosphorylated (Fig. 1a), acetylated (Fig. 1b) and double esterified starch (Fig. 1c)). This stronger signal is related to the stretching of the bonds between OH groups of starch and the H groups of the phenolic compounds, thus corroborating interaction of hydrogen bonds between polyphenolic compounds and wall materials. Moreover, it is possible to observe new intense signals at 1443, 1026 and 1618 cm^–1^, which are characteristic of the aromatic rings of polyphenolic compounds (*35*). Therefore, the latter can be associated with the presence of red sorghum phenolic compounds encapsulated in esterified starch; new signals also appear in the spectra of the three microcapsule treatments at 1620 and 1701 cm^–1^, corresponding to conjugated cycles (*35*). In addition, Gómez-Mascaraque *et al.* (*36*) reported a displacement of the 1042 cm^–1^ signal to 1038 and 1148 cm^–1^, as the evidence of microcapsules loaded with polyphenols, which coincides with the results obtained in this work.

#### Morphological properties of microcapsules

**Fig. 2:**
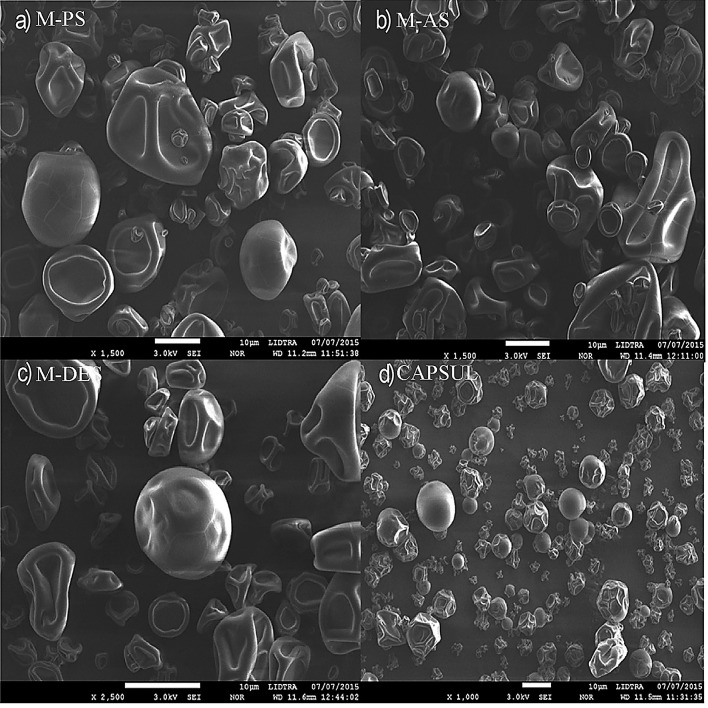
Scanning electron micrographs (SEM) of: a) microcapsules with phosphorylated starch (M-PS), b) microcapsules with acetylated starch (M-AS), c) microcapsules with double esterified starch (M-DES), and d) microcapsules with Capsul®

SEM micrographs in Fig. 2 show similar external morphologies of the microcapsules of phosphorylated, acetylated and double esterified starch and Capsul®, which revealed irregular shapes and particles with indented surfaces, obviating their agglomeration tendency, some of these with a rounded outer surface with some concavities. This feature is common in microcapsules dried by spray drying because of the rapid evaporation of water during the process. The average size of the microcapsules was not determined; however, the SEM micrographs allowed approximation of the size of the microcapsules to around 10 µm. Other authors have reported similar results (*37*, *38*).

#### Thermal properties of microcapsules

Endothermic transitions of microcapsules loaded with red sorghum polyphenols from phosphorylated, acetylated, double esterified starch and Capsul® were 173.89, 156.66, 144.08 and 141.93 °C, respectively (data not shown). The highest thermal stability was evident in microcapsules with phosphorylated starch. This could be explained by the fact that the phosphate group provides greater structural stability due to a higher molecular mass. The addition of high-molecular-mass groups into chemical structures increases endothermic transitions. These results are similar to those reported by Gonçalves da Rosa *et al.* (*35*) for microcapsules of gallic acid with chitosan as wall material.

### Stability of encapsulated phenolic compounds during storage

**Fig. 3:**
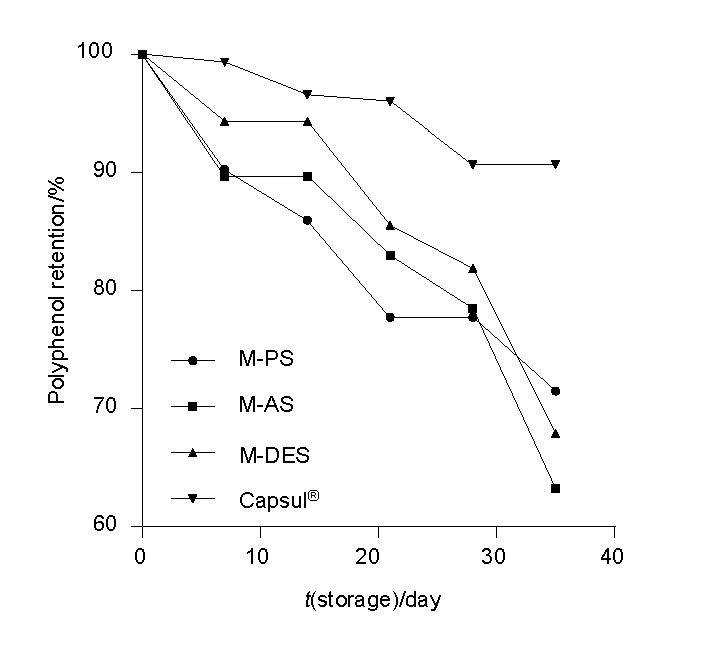
Stability of the microcapsules with red sorghum polyphenols during 35 days of storage at 60 °C: M-PS, M-AS and M-DES are microcapsules with phosphorylated, acetylated and double esterified starch, respectively

The microcapsule stability during storage at 60 °C is shown in Fig. 3. All of the microcapsules demonstrated a progressive decrease in polyphenolic content during storage. Retention of phenolic compounds after up to 28 days of storage was in the following order: microcapsules of double esterified starch>of acetylated starch>of phosphorylated starch. However, at the end of the 35-day period, microcapsules of phosphorylated starch maintained the highest polyphenol retention of more than 70%. Therefore, it can be deduced that the starch samples that contained the phosphate group in their molecular structure due to phosphorylation by extrusion, such as phosphorylated and double esterified starch, provided better stability of red sorghum phenolic compounds. This result is related to differential scanning calorimetry data (previously mentioned), in which phosphorylated starch was the wall material with greatest resistance to heat treatments, maintaining the structure. The high thermal stability of microcapsules of phosphorylated starch loaded with sorghum phenolic compounds can create new routes for its application in baked food products. Sáenz *et al.* (*39*) reported that indicaxanthins (betalain pigment) degraded slowly in all microcapsules during storage at 60 °C.

## CONCLUSIONS

In this research, we obtained a satisfactory encapsulation of the polyphenolic compounds of red sorghum using phosphorylated, acetylated and double esterified sorghum starch. The extrusion technique represents an attractive approach for obtaining esterified starch that can be applied as encapsulating material for the conservation of red sorghum phenolic compounds. To the best of our knowledge, the double esterification of sorghum starch has been successfully developed for the first time and evaluated as an encapsulating material. The introduction of acetyl and phosphate groups into the starch molecules was confirmed by FTIR analysis. Phosphorylated starch was the best cover material because it offered the greatest thermal stability compared with the other materials. Esterified sorghum starch as encapsulating agent by a spray dryer can improve the stability of sorghum phenolic compounds and optimize routes for various applications.
